# *Glycyrrhiza glabra* L. Saponins Modulate the Biophysical Properties of Bacterial Model Membranes and Affect Their Interactions with Tobramycin

**DOI:** 10.1021/acs.langmuir.5c00927

**Published:** 2025-04-30

**Authors:** Adam Grzywaczyk, Monika Rojewska, Wojciech Smułek, Daniel A. McNaughton, Krystyna Prochaska, Philip A. Gale, Ewa Kaczorek

**Affiliations:** † Institute of Chemical Technology and Engineering, Faculty of Chemical Technology, Poznan University of Technology, ul. Berdychowo 4, 60-965 Poznan, Poland; ‡ School of Mathematical and Physical Sciences, Faculty of Science, University of Technology Sydney, PO Box 123, Broadway, Sydney, NSW 2007, Australia; § School of Chemistry, The University of Sydney, Sydney, NSW 2006, Australia

## Abstract

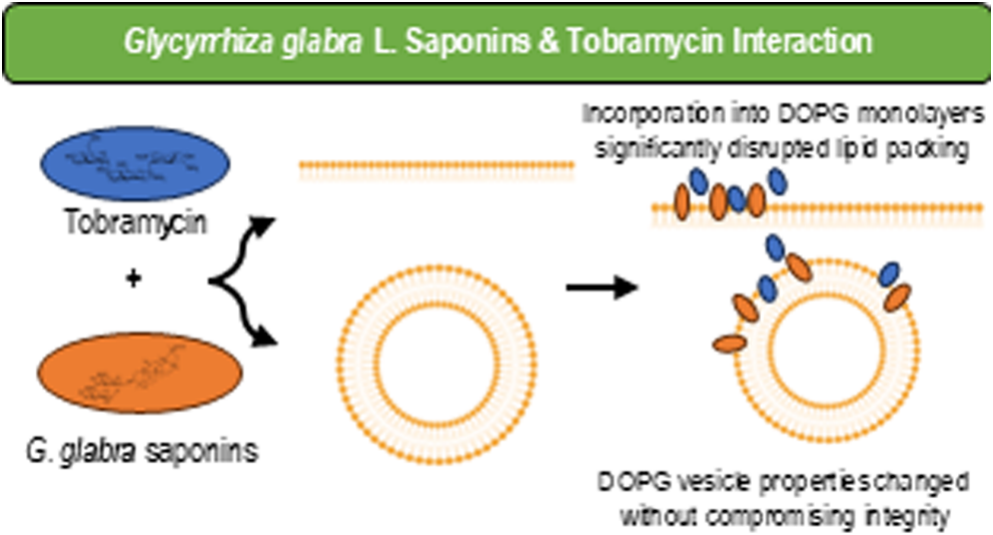

The global challenge of antibiotic resistance necessitates innovative approaches to improving the efficacy of existing therapeutics while mitigating their environmental impact. This study investigates the role of saponins derived from *Glycyrrhiza glabra* root extract in modulating interactions of tobramycin, a broad-spectrum aminoglycoside antibiotic, with model bacterial membranes composed of phosphatidylglycerol. Using Langmuir monolayers and vesicle models, we demonstrated that GgC saponins disrupt lipid packing, increasing membrane fluidity and altering biophysical properties. The addition of saponins at concentrations between 1.25 and 10 mg/L reduces the compressibility modulus of the lipid monolayer, with a decrease ranging from 25 to over 50%. ζ potential and dynamic light scattering analyses indicated that GgC–tobramycin interactions modify the surface charge without causing membrane lysis. These membrane changes could potentially facilitate enhanced interactions of antibiotics with bacterial cells. Importantly, these findings suggest the potential of natural surfactants such as saponins to improve antibiotic efficacy, possibly enabling reduced antibiotic dosages. This study provides insights into using saponins alongside antibiotics as a sustainable approach to addressing antibiotic resistance.

## Introduction

The escalating resistance of microorganisms to antibiotics is a critical global health threat, driven largely by their overuse in human medicine and agriculture.^1,2^ Overexposure accelerates the evolution of resistant bacteria, diminishing the effectiveness of the currently available antibacterial agents. With limited therapeutic options for resistant infections and a slow pace of new antibiotic development, there is an urgent need to explore alternative strategies. Addressing this global challenge requires a multifaceted approach, including improved surveillance, restricted antibiotic use, and collaborative action among national and international organizations.^1^ One promising approach within the realm of chemical research is the identification of adjuvants that can enhance the efficacy of existing antibiotics, potentially overcoming resistance, broadening the antibiotic spectrum, and reducing required dosages.^3^

Aminoglycosides, including tobramycin, are potent antibiotics effective against Gram-negative bacteria, particularly in severe infections.^4^ Despite declining use due to toxicity concerns, there is renewed interest in aminoglycosides to combat multidrug-resistant pathogens. Tobramycin, known for its broad-spectrum activity, is being explored as part of nanoparticle formulations that enhance efficacy and reduce side effects.^5^ To enhance its efficacy, tobramycin is also often used in combination with β-lactams or other cell-wall-disrupting agents to exploit synergistic effects and mitigate resistance development.^6−8^

Saponins, natural surfactants derived from plants, present a promising approach when used in tandem with traditional antibiotics. Saponins can insert into microbial cell membranes. Notably, bacterial membranes lack cholesterol (a major target of saponins in eukaryotic cell membranes); therefore, in bacteria, saponins likely interact with other lipid constituents. This interaction disrupts the membrane structure, leading to changes in its packing.^9^ By an increase in bacterial membrane fluidity, saponins may enhance membrane permeability, potentially facilitating antibiotic entry and their subsequent effects. For instance, saponins from *Sapindus mukorossi* L. significantly increased the antibacterial activity of nitrofurantoin against *Pseudomonas aeruginosa*.^7,8^ Similarly, glycyrrhizin (GC) acid enhanced the susceptibility of vancomycin-resistant enterococci to gentamicin, teicoplanin, and daptomycin.^10^ Interactions between saponins and model phospholipid membranes are important in the context of biogeochemical phospholipid cycles, which govern the movement and transformation of phospholipids in the environment. In natural bacterial ecosystems, changes in the composition and structure of lipid membranes can affect key biological processes, such as organic compound degradation and nutrient exchange, meaning saponins are particularly valuable in overcoming the protective barriers that resistant bacteria often employ. Using saponins to weaken the bacteria cell membrane could reduce the required dosage of antibiotics, decreasing the risk of side effects and slowing the development of resistance. Their natural origin and potential for synergy with antibiotics make them compelling candidates in the fight against antibiotic-resistant bacteria, offering a novel approach to enhance the efficacy of existing treatments.

In light of this, our study aimed to evaluate whether *Glycyrrhiza glabra* L. root extract (GgC) affects membrane properties that could influence tobramycin interactions with a model phospholipid membrane. The transportation of drugs across cell membranes is a complex and dynamic process, and model lipid membranes are invaluable tools when examining these processes. These models mimic key features of cellular lipids, enabling researchers to elucidate the roles of lipids in cellular interactions.^11^ Phospholipids are important constituents of all cell membranes. The chemical structure of individual phospholipids in the membrane is of significant importance because changes in the composition of acyl chains or headgroups affect the fluidity and stability of the bilayer and, consequently, affect the membrane’s response to external stimuli.^10−12^ The ability of bacteria to modify their phospholipid compositions leads to variations in the structure of their membranes. The most common phospholipids in bacterial membranes are phosphatidylglycerol (PG) and phosphatidylethanolamine (PE), which contain different polar headgroups. In our previous work, we have shown how saponins interact with model membranes composed of PE.^8,13^ This study examines interactions between saponins and antibiotics using a phosphatidylglycerol (DOPG) monolayer, aiming to determine whether saponins modulate membrane properties that are potentially relevant for antibiotic transport. The choice of DOPG, which is one of the predominant phospholipids found in bacterial membranes, provides a biologically relevant system to evaluate interactions between bacterial membranes, antibiotics, and saponins under controlled experimental conditions. We envisaged that this study would provide deeper insights into the mechanisms by which saponins can enhance the efficacy of antibiotics. Improving our understanding of how saponins interact with bacterial membranes, specifically through their impact on phospholipid composition and membrane fluidity, could help identify new ways to facilitate the penetration of antibiotics into bacterial cells. Advancements in this field have the potential to overcome one of the key barriers posed by resistant bacteria: their ability to modify membrane structures to impede drug entry. Additionally, these insights could reveal how saponins may disrupt bacterial defense mechanisms or synergize with antibiotics to improve treatment outcomes.

## Materials and Methods

### Chemicals and Membrane Model Structures Preparation

A chloroform of high-purity Uvasol (Merck, Warsaw, Poland) was used to prepare the Langmuir monolayer. Dulbecco’s phosphate buffered saline (Merck KGaA, Darmstadt, Germany) was applied as a solvent to prepare saponin solutions or as a subphase. 8-Hydroxypyrene-1,3,6-trisulfonic acid trisodium salt (HPTS) and 5(6)-carboxyfluorescein (5(6)-CF) were obtained from Sigma-Aldrich (Merck KGaA, Darmstadt, Germany). The saponins utilized in this study were obtained through a methanolic extraction process from *G. glabra* roots, employing a Soxhlet apparatus for 6 h. *G. glabra* extract is characterized by a complex chemical composition, which includes a variety of compounds, including flavonoids and polysaccharides. The key component of this extract is glycyrrhizic acid, which can vary in content from 2 to 25%.^14^ Tobramycin of 95% purity was obtained from Angene Chemical (Nanjing, Jiangsu, China).

Monolayer experiments were conducted by using the Langmuir technique. Two-dimensional (2D) models of biomembranes consist of DOPG (1,2-dioleoyl-*sn*-glycero-3-phospho-*rac*-(1-glycerol) sodium salt), 99%, from Avanti Polar Lipids (Alabaster, AL). Likewise, DOPG vesicles were prepared as liposomal bilayers (a three-dimensional membrane model—3D) via freeze–thaw and extrusion through a 200 nm porous membrane as described by Gilchrist et al.^15^

### Stability Measurements and Particle Size Distribution

To assess the stability of DOPG vesicles before and after treatment with tobramycin and GgC, we employed electrophoretic light scattering (ELS) to measure the ζ potential, utilizing the Smoluchowski equation for its determination. In this procedure, GgC, tobramycin, and their combination were added to 0.01% DOPG vesicles suspended in phosphate buffer solution (pH 7.0), achieving final concentrations of 5 mg/mL for tobramycin and 10 mg/mL for GgC. Additionally, the particle size distribution and polydispersity index (PDI) of the vesicles were assessed by using dynamic light scattering (DLS). Both analyses were conducted using a Litesizer 500 instrument (Anton Paar, Graz, Austria).

### HPTS and 5(6)-Carboxyfluorescein

To evaluate the potential leakage and damage to vesicles upon treatment with tobramycin and GgC, separate experiments were completed with either HPTS or 5(6)-CF dyes encapsulated within the liposomes. This was achieved by using aqueous solutions as the rehydration medium for the phospholipid film before freeze–thawing, following the method described by Gilchrist et al.^15^ After the freeze–thaw process, the liposomes were extruded through a 200 nm membrane before being separated from the unencapsulated dye using Sephadex size exclusion chromatography (SEC). This was achieved using specific buffered solutions as the rehydration medium. For the HPTS assay, the internal liposome solution was 100 mM NaCl, 10 mM *N*-(2-hydroxyethyl)piperazine-*N*′-ethanesulfonic acid (HEPES), 1 mM HPTS (pH 7.2), and after SEC purification, the external buffer was 100 mM NaCl, 10 mM HEPES. For the 5(6)-carboxyfluorescein assay, the internal solution was 451 mM NaCl, 20 mM phosphate buffer (pH 7.2) with 50 mM 5(6)-carboxyfluorescein, and the external solution after SEC was 150 mM Na_2_SO_4_, 20 mM phosphate (pH 7.2). The liposomes, containing 0.1% of the dye, were subsequently treated with tobramycin and GgC, reaching final concentrations of 5 and 10 mg/mL, respectively. Triton-X100 was added at the end of the experiment to lyse the liposomes and determine the maximum possible fluorescence, serving as a reference for total leakage. Fluorescence emission was monitored in real-time using the GloMax Explorer System (Promega).

### π–*A* Isotherm Measurement

All of the experiments were performed using the KSV NIMA Langmuir film balance system (KN 0033). The surface pressure was measured using a Wilhelmy platinum plate with an accuracy of ±0.1 mN/m. The lipid monolayer (DOPG) was formed by dropping 25 μL of phospholipid solution onto a subphase interface. As the subphase, ultrapure water with a conductivity of 0.055 μS/cm and a pH of 6.7 was used (PureLab System, ELGA, Poland). The subphase was placed in a Teflon trough (KSV Nima, Helsinki, Finland) with a surface area of 238 cm^2^. During the measurements, the temperature was kept constant at 25.0 ± 0.1 °C with a Julabo circulator. Before each measurement, the subphase was cleaned to a surface pressure below π = 0.35 mN/m reached at maximum compression. After spreading the phospholipid solution onto the subphase, it was followed by 15 min evaporation of the chloroform, the Langmuir monolayer was compressed at a constant barrier speed equal to 10 mm/min. One minute before the compression, the saponin solution was injected into the Langmuir trough and stirred. Saponin solution (10 mg/mL) was added in an appropriate volume to achieve the following final subphase concentrations: 1.0; 2.5; 5.0, and 10.0 mg/L. The surface pressure π (mN/m) was measured as a function of the area per DOPG molecule, *A* (Å^2^/molec.).

A compressional modulus (*C*_s_^–1^) is a rheological quantity related to monolayer rigidity, and it is calculated from the π*–A* isotherm data using eq 1:
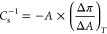
1The magnitude of this parameter provides information about the monolayer packing and the elasticity of the monolayer during compression. According to the Davies and Rideal classification,^15^ the values of 12.5 < *C*_s_^–1^ < 50 mN/m and 50 < *C*_s_^–1^ < 250 mN/m indicate that the monolayer is formed in a liquid-expanded (LE) state and liquid-condensed (LC) state, respectively. Each experiment was repeated at least three times to ensure that the reproducibility of the curves was within ±2 Å^2^.

### Relaxation Measurements

To introduce additional molecules into the monolayer using a peristaltic pump, the monolayer was formed first, and then the subphase was replaced with a new solution. The exchange was performed through silicone tubing placed on a stand before the inlet and outlet hoses were inserted into the Langmuir trough on opposite sides. Both hoses were connected to the same pump head, which ensured a steady supply and the removal of liquid from the trough. All relaxation experiments described in this article were carried out with subphase exchange using a MINIPULS 3 (Gilson) peristaltic pump.

### Statistical Analysis

Statistical analyses were performed using Python (v3.9) with the *statsmodels* and *seaborn* libraries. Data preparation involved simulating distributions based on the provided means and standard deviations for each treatment group. Normality was assessed using histograms and Q–Q plots, while Levene’s test was applied to evaluate the homogeneity of variances. One-way analysis of variance (ANOVA) was conducted to determine the effect of the treatments. Post hoc comparisons were performed using Tukey’s Honest significant difference (HSD) test to identify pairwise differences between groups.

## Results and Discussion

### *Glycyrrhizin* Saponins and Tobramycin Effect on DOPG Vesicle

Initial experiments focused on evaluating the effects of *G. glabra* saponins and tobramycin on DOPG vesicles in order to assess how these compounds influence vesicle stability, size distribution, and surface charge. Concentrations of 5 mg/mL for tobramycin and 10 mg/mL for GgC were chosen to align with typical therapeutic dosages, ensuring relevance to clinical applications.

**Figure 1:**
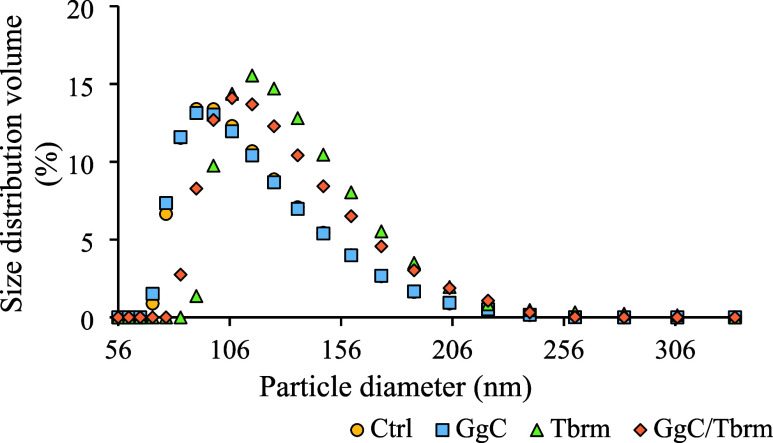
*G. glabra* L. root extract (GgC) and tobramycin affect the vesicle size distribution. Ctrl—untreated sample was treated with GgC (5 mg/L), Tbrm (10 mg/L) with tobramycin, GgC/Tbrm (5 mg/L) with GgC (10 mg/L) with tobramycin effect. The *p*-value is greater than 0.05, indicating that variances across groups are not significantly different.

The control sample, consisting of untreated DOPG liposomes, exhibited a narrow size distribution with a peak particle diameter centered around 100–120 nm (Figure 1), indicating a uniform population with minimal size variability. Treatment with GgC extract maintained the size distribution peak at 100–120 nm, similar to the control. However, a slight broadening of the distribution was observed (Figure 1), suggesting an increase in size variability among the liposomes. This implies that while GgC slightly affects the homogeneity of liposome sizes, it does not significantly alter their overall mean size. In contrast, liposomes treated with tobramycin exhibited a peak size distribution shifted toward larger diameters (115–130 nm) compared to both the control and GgC-treated samples. The distribution also appeared broader than that of the control, indicating an increased heterogeneity. This suggests that tobramycin induces mild structural changes in the liposomes, contributing to the size variability. When GgC and tobramycin were combined, the size distribution remained centered around 100–120 nm but exhibited a more pronounced broadening and a slight shift toward larger diameters compared to the individual treatments. This broader distribution suggests an interaction between GgC and tobramycin, likely enhancing structural alterations in the liposomes and potentially leading to aggregation, or GgC may inhibit the structural alterations caused by the antibiotic. Volume-weighted size distributions are reported here to provide a representative vesicle size profile that is less biased by very large or small particles. For completeness, intensity-weighted and number-weighted distributions are included in the Supporting Information, showing similar trends. Despite these observed shifts, the polydispersity index (PDI) values (Table 1) remained low across all samples, confirming a generally uniform size distribution under all conditions. The combination of GgC and tobramycin, however, caused subtle changes in the distribution peak and variability, indicating enhanced structural effects on the liposome population.

**Table 1:** Polydispersity Index (PDI) of Samples^a^^,^^b^

Ctrl	0.13 ± 0.02*
GgC	0.15 ± 0.02*
Tbrm	0.13 ± 0.03*
GgC/Tbrm	0.13 ± 0.04*

a Ctrl—untreated sample, GgC—5 mg/L treated, Tbrm—10 mg/L tobramycin, GgC/Tbrm 5 mg/L, GgC + 10 mg/L of tobramycin effect.

b One-way ANOVA indicated no significant differences among the PDI (*: *p* > 0.05).

**Figure 2:**
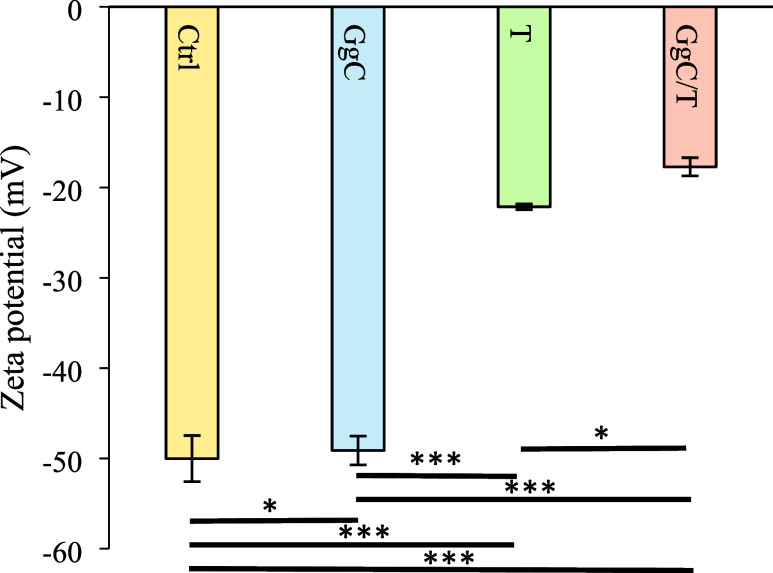
ζ potential of DOPG liposomes under different conditions (Ctrl: untreated control; GgC: *G. glabra* crude saponin extract at 5 mg/L; T: tobramycin at 10 mg/L; GgC/T: combination of GgC + tobramycin). Asterisks indicate statistically significant differences: *: *p* < 0.05, ***: *p* < 0.001.

The ζ potential of untreated DOPG liposomes was measured at −50.0 mV, reflecting a strongly negative surface charge (Figure 2). This high level of negative charge indicates that the control DOPG liposomes have a strongly charged surface with significant electrostatic repulsion between vesicles. Upon treatment with GgC, the zeta potential showed minimal influence on the stability of the liposomes (within experimental error). The marginal decrease in surface charge suggests that the electrostatic repulsion remains largely comparable to that of the untreated liposomes. In contrast, treatment with tobramycin led to a significant decrease in ζ potential, recorded at −22.1 mV. This substantial reduction in surface charge indicates that tobramycin compromises the stability of the DOPG vesicles, likely by neutralizing some of the negative charges on the liposome surface. The resulting decrease in repulsive forces increases the likelihood of liposome aggregation. When the liposomes were exposed to both GgC and tobramycin, the ζ potential further decreased to −17.7 mV. This additional reduction in surface charge, compared to that of tobramycin alone, suggests that the combination of saponin extract and tobramycin severely undermines liposome stability. The interaction between these compounds appears to further neutralize the liposome surface charge, making the liposomes even more prone to aggregation.

**Figure 3:**
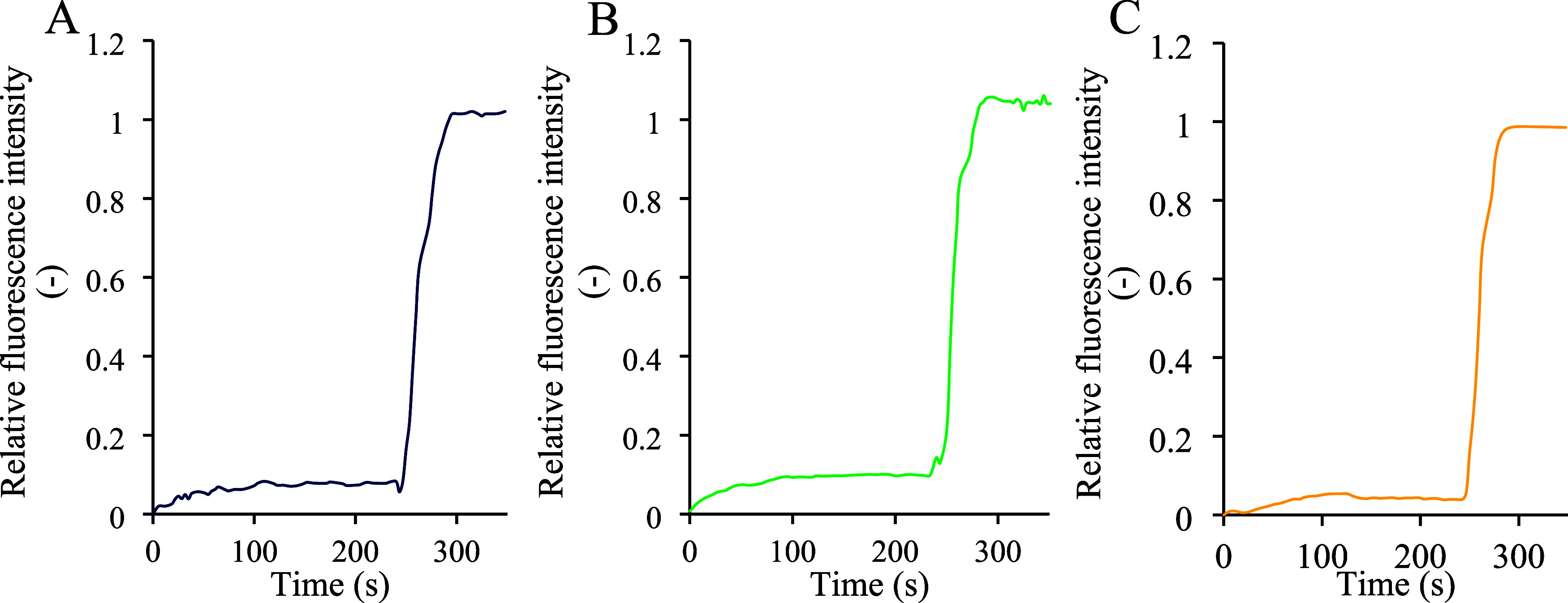
HPTS release assay; (A) GgC 5 mg/L treatment; (B) tobramycin 10 mg/L effect; and (C) tobramycin and *Glycyrrhiza* extract action. After 210 s, liposomes were lysed by adding a surfactant to obtain the maximum fluorescence signal.

The stability of DOPG liposomes treated with *GgC* tobramycin and their combination was assessed using two complementary methods: the HPTS assay and the carboxyfluorescein release assay. These techniques provide a comprehensive evaluation of the liposome stability and membrane permeability. The HPTS assay monitors internal pH changes, which reflect ion leakage and membrane transport, while the carboxyfluorescein assay measures the release of an encapsulated dye, directly assessing membrane integrity. By using both methods, we can gain valuable insights into the effects of GgC, tobramycin, and their combination on the structural stability and ion permeability of the liposomal membrane. In the HPTS assay, the stability of the liposomes was tracked by measuring the internal pH changes. Before the addition of Triton-X100, liposomes treated with GgC resulted in little change in fluorescent output, indicating a minimal passage of H^+^ into the membrane, and therefore preservation of integrity (Figure 3). Similarly, tobramycin-treated liposomes and liposomes exposed to the combination of GgC and tobramycin exhibited stable fluorescence, demonstrating that neither the antibiotic alone nor the combination of GgC and tobramycin destabilized the membrane. Overall, these results indicate that before the detergent lysis step, none of the treatments (GgC, tobramycin, or their combination) caused significant ion leakage or compromised the liposome membrane integrity.

**Figure 4:**
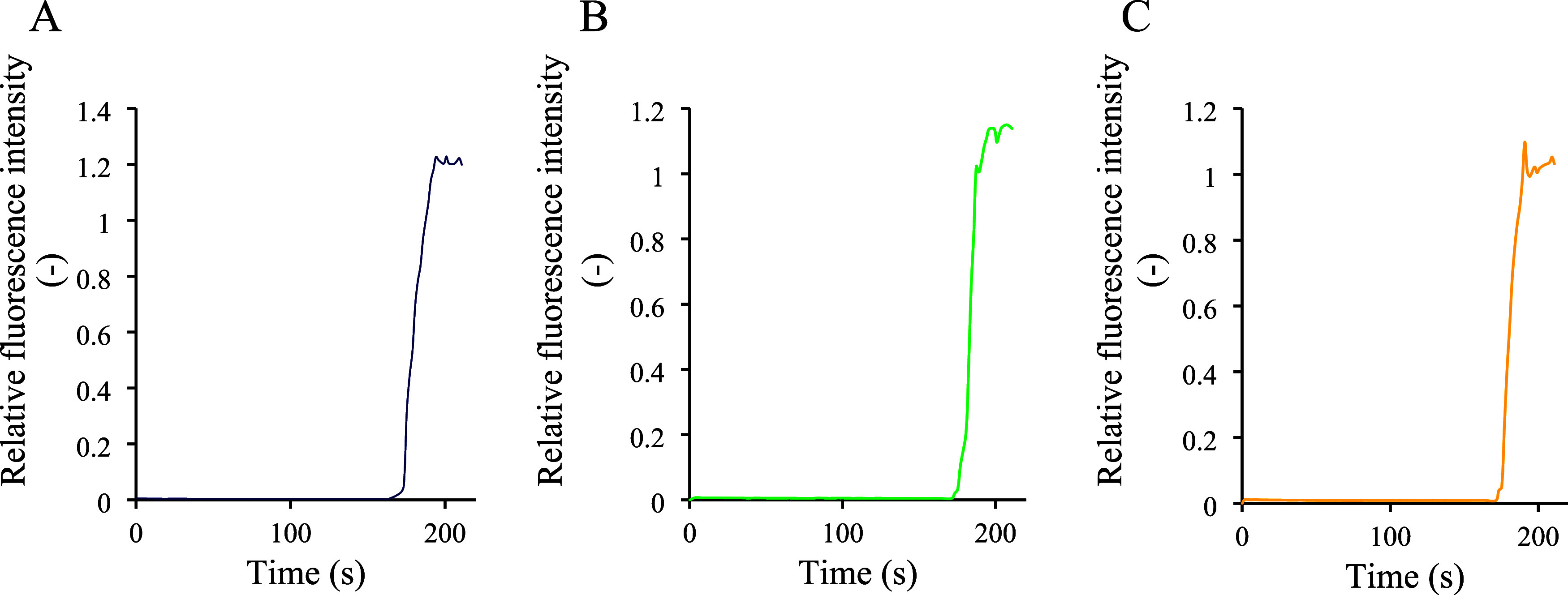
CF release assay: (A) GgC treatment; (B) tobramycin effect; and (C) tobramycin and *Glycyrrhiza* extract. After 175 s, the destruction of liposomes was forced by the addition of surfactant.

The carboxyfluorescein release assay provided further confirmation of membrane stability by measuring the release of the encapsulated dye (Figure 4). As in the HPTS assay, Triton-X100 was added at the end to lyse the liposomes and establish maximum fluorescence. Liposomes treated with GgC, tobramycin, and their combination did not exhibit a change in fluorescence before Triton-X100 addition, indicating minimal dye leakage and intact membranes. Importantly, the combination of GgC and tobramycin also did not result in membrane rupture. Together, the results of the HPTS and carboxyfluorescein assays provide comprehensive evidence that DOPG liposomes remain structurally intact and stable when exposed to GgC, tobramycin, and their combination.

### Impact of GgC Saponins Extract and Antibiotic on the DOPG Monolayer

The second phase of the study explored the impact of GgC and tobramycin on the DOPG monolayer, a model system mimicking bacterial membranes. Higher concentrations of both GgC and tobramycin were used in this stage to thoroughly examine their influence on the structural properties of the lipid monolayer. The goal was to assess how these compounds affect membrane packing, surface pressure, and fluidity, providing insight into their potential to enhance antibiotic penetration.

**Figure 5:**
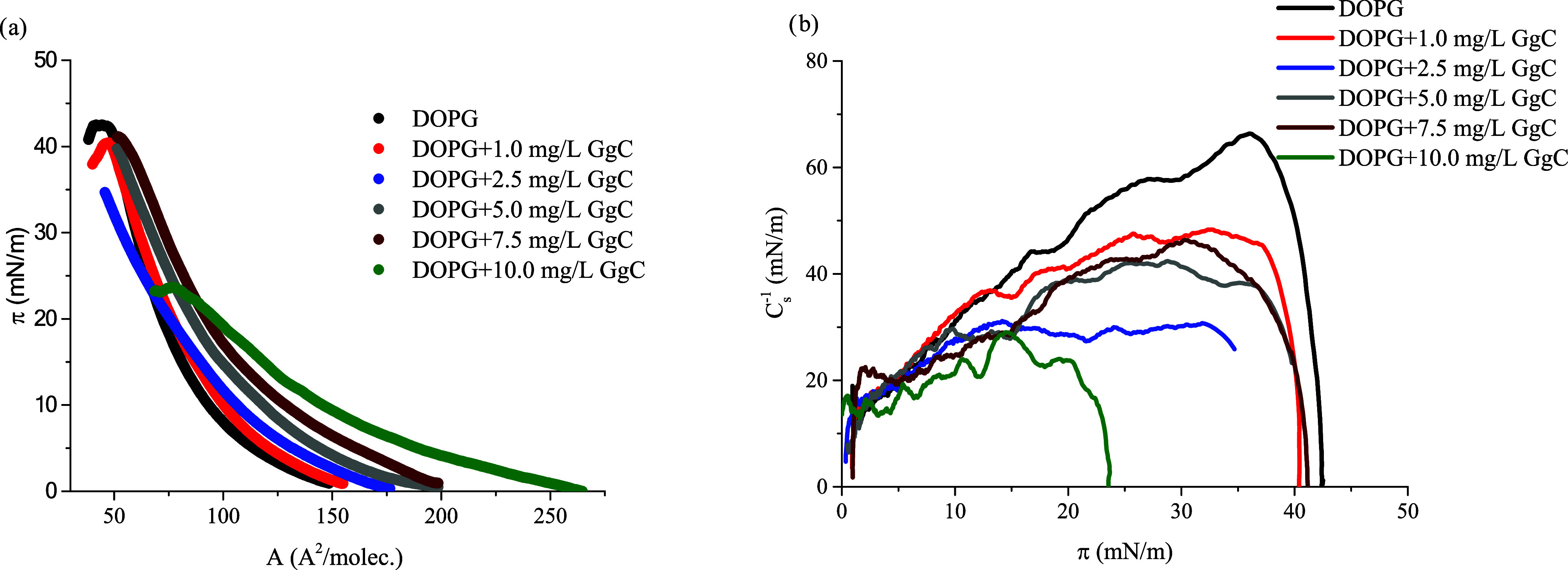
Surface pressure–area per molecule isotherms (π–*A*): (a) compression modulus–area per molecule (*C*_s_^–1^–π), (b) for the analyzed system: DOPG with various concentrations of the crude saponins extract GgC.

Figure 5 illustrates the surface pressure–area per molecule (π–*A*) isotherms obtained for the DOPG monolayer with different concentrations of saponin extract in the bulk subphase. For all analyzed systems, the π–*A* isotherms are shifted markedly to larger molecular areas relative to the pure DOPG monolayer. The lift-off area, *A*_lift-off_, defines the area per molecule at which the isotherm begins to rise, indicative of the onset of intermolecular interactions. The *A*_lift-off_ values increase with a higher concentration of saponins in the system. We have shown in our previous research^9^ that expanded lipids film is caused by interactions of saponin molecules with phospholipids.

The obtained *A*_lift-off_ values for systems DOPG + GgC are higher than those for a pure DOPG monolayer. Therefore, one can expect that saponin molecules are incorporated into the lipid structure and strongly expand the phospholipid membrane. Table 2 gives detailed information about the π–*A* isotherm parameters.

**Table 2:** Characteristic Parameters of π–*A* Isotherms^a^^,^^b^

	*A*_lift-off_ (Å^2^/molec.)	*A*_collapse/maximum_ (Å^2^/molec.)	π_collapse/maximum_ (mN/m)	*C*_s,max_^–1^ (mN/m)
DOPG	137.1	50.4	43.2	66.4
DOPG + 1.25 mg/L GgC	145.3	55.3	41.0	50.3
DOPG + 2.5 mg/L GgC	170.6	50.0	34.3	31.1
DOPG + 5.0 mg/L GgC	190.2	62.1	39.1	42.2
DOPG + 10.0 mg/L GgC	245.0	80.1	24.2	30.7

a *A*_lift-off_—lift-off area of surface pressure. *A*_collapse/maximum_—area corresponding to the monolayer collapse or maximum surface pressure, π_collapse/maximum_—collapse pressure or maximum pressure reached by compressing [mN/m], max. *C*_s_^–1^—maximum value of the compression modulus [mN/m], phosphatidylglycerol (DOPG).

b *A*_lift-off_ values for DOPG film are ca. 137 Å^2^/molec., while the addition of 10 mg/L GgC extract to subphase caused a nearly 2-fold increase compared to the *A*_lift-off_ for DOPG monolayers only. This effect may be a consequence of the formation of aggregates, which can occupy a much larger surface area at the interface than a single phospholipid molecule. Moreover, the addition of a higher concentration of GgC extract changes the inclination angle of the π–*A* curve, which also confirms the interactions between saponins and phospholipid molecules.

There is a smaller increase in the surface pressure within the monolayer compression for higher saponin concentrations in the subphase. Although the observed effects vary somewhat with concentration, at higher concentrations, a strong steric crowding effect of molecules may occur. Moreover, for some considered systems, a high extract concentration leads to the collapse of the monolayer at low surface pressure (π_collapse_). At the collapse pressure, the Langmuir monolayer undergoes a phase transition from a two-dimensional (2D) fluid into the subphase to a three-dimensional (3D) bulk phase. The collapse or maximum surface pressure point is reached at the lower surface pressure (π_collapse_ ca. 24 mN/m) and the greater area occupied by molecules at the interface. These results suggest that a higher concentration of GgC decreases the stability of the DOPG film and enhances its structural deformation.

The π–*A* (surface pressure–molecular area) isotherm typically possesses distinct regions that correspond to different molecular packing arrangements in different surface pressure regimes. The run of π–*A* isotherm for DOPG is consistent with the literature data.^16^ The DOPG monolayer with saponins forms only a liquid-expanded phase (LE) according to the estimated maximum *C*_s_^–1^ value.^17^ If the surface pressure increased, then the *C*_s,max_^–1^ value also became greater and reached ca. 66 mN/m. This phenomenon is particularly visible when analyzing the behavior of lipid layers in the presence of 10 mg/L saponin extract. The collapse surface pressure value for DOPG + 10 mg/L GgC is ca. 24 mN/m. Films formed by DOPG and GgC extract molecules characterized a compression modulus value lower than that for the DOPG monolayer. Generally, the obtained *C*_s,max_^–1^ value decreases with the rise of the extract concentration in the subphase. The formed mixed monolayers DOPG + GgC refer to the LE state. The findings demonstrate that saponins incorporated into the phospholipid monolayers cause strong fluidization. Likely, only the hydrophilic sugar part is generally submerged in the aqueous phase, while the aglycone part extends toward the air.^18^ This effect was observed for other systems’ lipid monolayer-saponins.^9,17^ Moreover, Krochowiec et al.^19^ have shown that the fluidizing effect was more significant in the case of saponins bearing a large aglycone moiety.

Based on our previous results,^12^ we also state that the appropriate concentrations of saponin extract could improve the penetration of the antibiotic molecules through the lipid bilayer of the bacterial membrane. Our results allow us to assume that the concentrations of GgC saponins strongly impact the structure and packing of lipid monolayers.

In the next step, the impact of tobramycin on the surface properties of the DOPG monolayer was analyzed.

**Figure 6:**
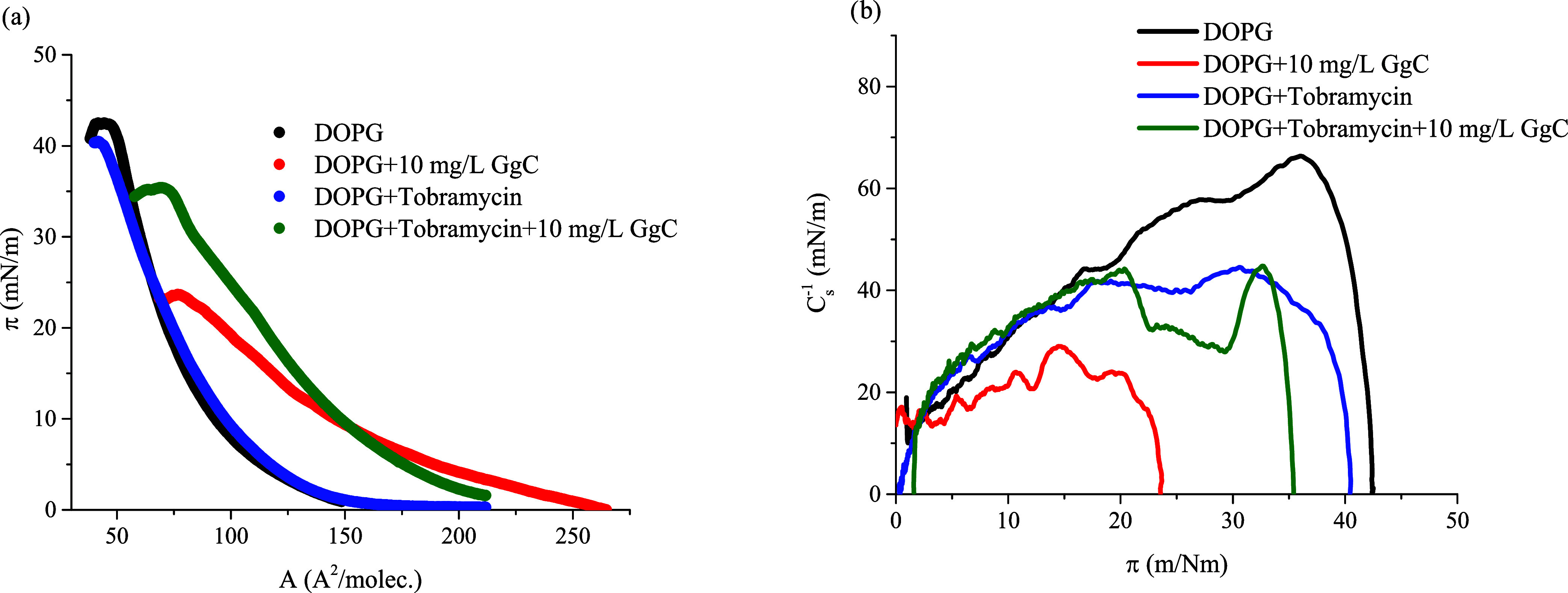
Surface pressure–area per molecule (π–*A*) isotherms: (a) compression modulus–area per molecule (*C*_s_^–1^–π), (b) for the analyzed systems: DOPG with the crude saponins extract GgC and antibiotic, phosphatidylglycerol (DOPG).

1 mL of antibiotic solution was added to the subphases, resulting in the final concentration of 5 mg/L. At the beginning of compression, the injected tobramycin does not strongly interact with the DOPG monolayers (blue line, Figure 6a). Practically, the run of π–*A* isotherm for pure DOPG and DOPG + tobramycin is similar. However, the *C*_s_^–1^ parameter obtained for DOPG + tobramycin for strong compression is much lower than that for a pure DOPG monolayer. These data showed that tobramycin can alter the elastic properties of DOPG lipid monolayer by decreasing the cohesion between DOPG molecules. A similar effect was observed by Fa and co-workers^20^ who studied interactions between azithromycin with 1,2-dioleoyl-*sn*-glycero-3-phosphocholine (DOPC) bilayers. The characteristic parameters of the π–*A* isotherms are listed in Table 3.

**Table 3:** Characteristic Parameters of π–*A* Isotherms^a^

	*A*_lift-off_ (Å^2^/molec.)	*A*_collapse/maximum_ (Å^2^/molec.)	π_collapse/maximum_ (mN/m)	*C*_s,max_^–1^ (mN/m)
DOPG + tobramycin	163.1	42.3	40.2	46.8
DOPG + tobramycin + 10 mg/L GgC	200.3	72.4	35.6	45.4

a *A*_lift-off_—lift-off area of surface pressure. *A*_collapse/maximum_—area corresponding to the monolayer collapse or maximum surface pressure, π_collapse/maximum_—collapse pressure or maximum pressure reached by compressing (mN/m), max. *C*_s,max_^–1^—maximum value of the compression modulus (mN/m), phosphatidylglycerol (DOPG).

A clear difference in the course of the π–*A* isotherm was obtained for the DOPG + tobramycin + GgC system. The presence of tobramycin and saponins in the subphase impacts the interactions between molecules at the interface, which is demonstrated by a greater increase in the surface pressure (π) during compression of the DOPG + tobramycin + GgC monolayer. Adding tobramycin to the subphase changes the character of interactions; the mixed monolayer of these three components is characterized by higher compressibility and elasticity. In consequence, the DOPG + tobramycin + GgC monolayer reaches higher surface pressure values than the DOPG + GgC system (Table 3). As shown in Figure 6, tobramycin molecules do not strongly interact with the DOPG monolayer; the additional compressibility in the mixed system is likely determined by interactions between the antibiotics and saponin molecules.

These studies were followed by an experiment examining the relaxation process of the DOPG monolayer in the presence of GgC extract and an antibiotic in the subphase. For this purpose, the DOPG film was compressed to the surface pressure of 30 mN/m, and after compression, the surface per molecule was estimated as *A*_0_. At this surface pressure, the lipid packing density is similar to that of a cell membrane (pure DOPG), and the lipid monolayer mimics the outer surface of a cell membrane. By keeping the film area constant, changes in surface pressure are recorded upon addition of drugs to the subphase. Therefore, when injecting saponin extract into the subphase, a change in surface value per molecule in the DOPG monolayer (*A*(*t*)) is observed. The increase in (*A*(*t*)) compared to *A*_0_ indicates the increase in the area per molecule caused by the incorporated saponin molecules into the phospholipid monolayer. Otherwise, if *A*(*t*)/*A*_0_ < 1, there is a surface area loss in the monolayer, which could result in their desorption from the monolayer to the subphase. Consequently, the monolayer stability can be estimated as the *A*/*A*_0_ parameter.

**Figure 7:**
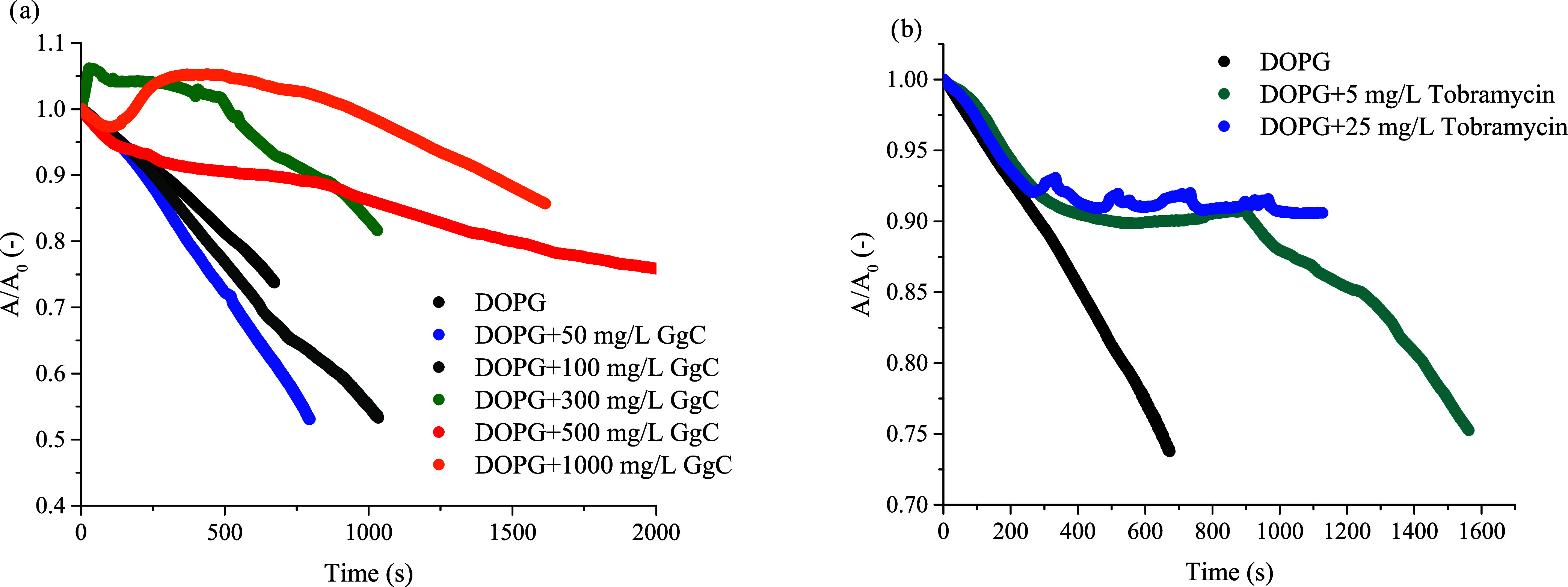
Relative area–time curves for the DOPG monolayer in the control sample (with buffer only) and (a) with saponins injected into the subphase in concentrations 50, 100, 300, 500, and 1000 (mg/L) and (b) with tobramycin in concentrations 5 and 25 mg/L. The plot shows the normalized area per molecule (*A*/*A*_0_) as a function of time, where *A*_0_ = *A* (for *t* = 0), phosphatidylglycerol (DOPG).

No interaction effect of saponins with the DOPG monolayer was observed for their low concentrations in the subphase, 5 or 10 mg/L. Therefore, saponin extract was added across a concentration range of 50–1000 mg/L in the subphase to determine the concentration when molecules are absorbed into the lipid monolayer. Figure 7a shows how the stability of the DOPG monolayer depends on saponin concentration and that the incorporation of saponin molecules into the DOPG film is only visible for higher concentrations of extract injected into the subphase. For concentrations greater than 300 mg/L, the relaxation curves changed the run *A*/*A*_0_ = *f*(*t*) in comparison to that of the pure DOPG monolayer. The addition of saponin improved the stability of the DOPG monolayer, indicating that interactions between the saponin present in the subphase and the lipid polar headgroups lead to some conformational disordering of the lipid molecules and formation of a more stable mixed monolayer.

Figure 7b shows the behavior of the DOPG monolayer in the presence of tobramycin. The injection of antibiotics into the subphase altered the relaxation behavior of the lipid film. After a maximum was reached, the normalized area *A*(*t*)/*A*_0_ gradually decreased below 1.0, indicating a net loss of monolayer area over time. This could be due to a portion of the adsorbed saponin leaving the interface (desorbing) or due to molecular rearrangements that reduce the occupied area. The pure DOPG monolayer shows a significant decrease in *A*/*A*_0_ after 500 s, which corresponds to a 25% decrease in area. In contrast, for the DOPG + 25 mg/L tobramycin system, only a 10% decrease in area is observed. Moreover, the higher concentration of tobramycin added resulted in the formation of a more stable mixed film.

**Figure 8:**
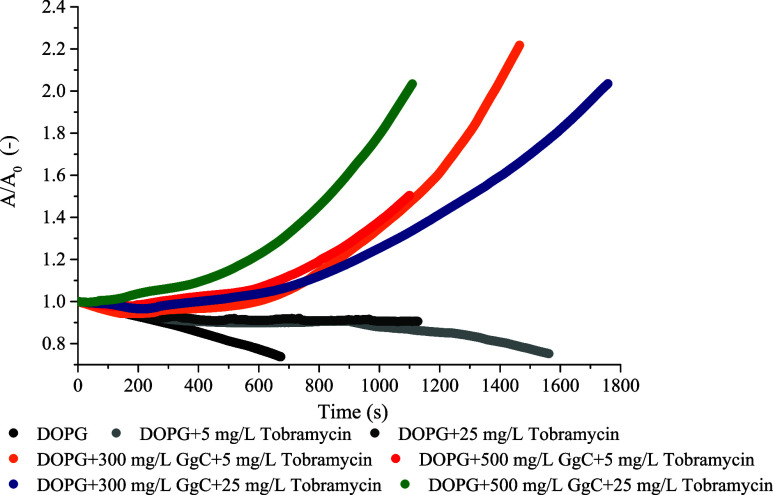
Relative area–time curves for the DOPG monolayer in the control sample (with buffer only) and with saponins injected into the subphase in concentrations: 300, 500 mg/L, and/or with tobramycin in concentrations: 5 and 25 mg/L. The plot shows the normalized area per molecule (*A*/*A*_0_) as a function of time, where *A*_0_ = *A* (for *t* = 0), phosphatidylglycerol (DOPG).

The impact of mixtures of GgC extract and antibiotic on the model lipid membrane is shown in Figure 8. The relaxation of monolayers was registered for saponin extract at concentrations: 300 and 500 mg/L and tobramycin: 5 or 25 mg/L. A mixture of saponins and antibiotics was injected into the buffer subphase beneath the DOPG monolayer.

Generally, for all considered systems of DOPG + tobramycin + GgC, an expanding film at the interface was observed. The increasing relative area for molecules at the interface indicates that saponins and tobramycin strongly interact with the phospholipid monolayer and are incorporated into the membrane structure. The introduction of the saponin extract into the subphase results in increased membrane expansion due to its amphiphilic nature, which disrupts lipid packing and enhances lipid mobility. The surface area doubles after 15 min in a system with the addition of 500 mg/L GgC and 25 mg/L tobramycin. A similar tendency was observed for 300 mg/L GgC, but the adsorption process took more time. By comparing the results for the GgC + tobramycin mixture (Figure 8) with the obtained relaxation curves for component systems (Figure 7a,b), it can be concluded that the simultaneous addition of saponin and antibiotic had a more pronounced expanding effect on the DOPG film than either agent alone. This suggests a potentially cooperative interaction between GgC and tobramycin in disrupting the membrane.

The incorporation of GgC into DOPG monolayers significantly increased the molecular area per lipid, indicating that saponin inserts into the film and expands the lipid packing. Moreover, the GgC-induced reduction in the monolayer’s compressional modulus (greater compressibility) and the decrease in collapse pressure point to a more fluid, less rigid film in the presence of saponin. This expansion effect, evidenced by shifts in the π–*A* isotherms, aligns with previous research highlighting saponins’ capacity to enhance membrane permeability and reduce lipid rigidity. Glycyrrhetinic acid (GA), the aglycon of glycyrrhizin (GC), was found to disrupt lipid raft models by reducing domain size and promoting fluid networks.^21^ At higher concentrations, GgC reduced the collapse surface pressure (π_collapse_) and compressional modulus (*C*_s_^–1^), confirming its membrane-destabilizing and fluidizing properties. The incorporation of GgC into DOPG monolayers at the air–liquid interface was marked by a significant shift in the π–*A* isotherms toward larger molecular areas. For instance, the area per molecule increased from 137 Å^2^ for pure DOPG to 245 Å^2^ when 10 mg/L GgC was added, indicating disruption of lipid packing. This aligns with our previous studies demonstrating that some saponins expand lipid bilayers and reduce membrane rigidity.^9,12,18,19^ Additionally, glycyrrhizin, a key bioactive component of *G. glabra*, has been shown to integrate into DOPG vesicles, modifying their structure while enhancing stability.^22^ At higher GgC concentrations, the reduction in π_collapse_ and *C*_s_^–1^ values further validated its role in destabilizing the monolayer and promoting fluidity. These findings align with reports of saponins reducing lipid packing density to enhance membrane fluidity and disrupt structural integrity.^23,24^ Tobramycin alone, however, exhibited minimal interaction with the DOPG monolayer, as evidenced by only slight reductions in the level of *C*_s_^–1^. This suggests weak interactions between tobramycin and the lipid layer, consistent with its known reliance on active transport mechanisms for bacterial cell penetration.^25^ When combined with GgC, notable changes in the π–*A* isotherms were observed, characterized by a decreased surface pressure and slightly lower monolayer compressibility, suggesting that GgC increases the membrane fluidity. Such an increased fluidity seems to facilitate interactions between tobramycin and the lipid membrane.

The combined use of saponins and antibiotics has been previously investigated, with some saponins shown to alter bacterial membrane properties.^19,26,27^ ζ potential measurements revealed a progressive increase (less negative values) following treatment with GgC, tobramycin, and their combination, indicative of alterations in the liposome surface charge. Positively charged tobramycin interacts electrostatically with negatively charged phospholipid headgroups of DOPG, partially neutralizing the surface charge.^28,29^ When GgC was combined with tobramycin, a more pronounced reduction in the surface charge was observed. This may result from additional interactions at the lipid–water interface facilitated by the amphiphilic nature of saponins. The hydrophilic sugar moieties of saponins could partially mask negative charges on the liposome surface, while the lipophilic aglycone regions insert into or associate with the lipid bilayer.^9^ Despite these surface interactions, dynamic light scattering (DLS) data indicated no significant detrimental effect on particle size or polydispersity (PDI), suggesting that the liposome population remained uniform and stable under the studied conditions. While some reports have noted vesicle disruption and fusion in the presence of saponins,^22,23^ our membrane integrity assays (HPTS and carboxyfluorescein leakage) demonstrated minimal membrane disruption, indicating vesicles remained structurally intact under the tested conditions.

Collectively, these observations demonstrate that *G. glabra* saponins modify biophysical membrane properties, potentially affecting antibiotic–membrane interactions. Although enhanced fluidity and reduced membrane rigidity suggest conditions favorable for antibiotic entry, direct evidence of increased tobramycin penetration into membranes was not obtained in this study. Further direct transport assays would be necessary to confirm such antibiotic uptake. Nonetheless, the observed biophysical membrane modulation is consistent with previous studies highlighting saponin-mediated effects that could aid antibiotic action.^10,26,28,30,31^

## Conclusions

This study highlights that *G. glabra* L. saponins (GgC) modify the biophysical properties of bacterial model membranes, influencing their interaction with tobramycin. GgC incorporation into DOPG monolayers significantly disrupted lipid packing, altering membrane properties, such as molecular area and compressibility. The combination of GgC and tobramycin led to changes in the vesicle surface charge and size distribution but did not compromise membrane integrity. The combined presence of GgC and tobramycin showed an enhanced effect on the membrane fluidity (relative to each alone). These biophysical modifications suggest a potential for GgC to enhance antibiotic interactions with bacterial membranes, offering a promising strategy to address antibiotic resistance through sustainable coformulations. Further research is warranted to validate these findings across different bacterial systems and antibiotics in clinical and environmental settings.

## Supporting Information Available

Dynamic light scattering (DLS) analysis presented in three distinct weighting models: number, volume, and intensity (PDF)
