# Supplementary material for: Glycyrrhiza glabra L. Saponins Modulate the Biophysical Properties of Bacterial Model Membranes and Affect Their Interactions with Tobramycin
Source: Langmuir. 2025 Apr 30;41(18):11701–10. doi: 10.1021/acs.langmuir.5c00927 (PMC12080323; doi:10.1021/acs.langmuir.5c00927)
Supplement: Supplementary file 1 — la5c00927_si_001.pdf [file la5c00927_si_001.pdf]

Supplementary Information

Glycyrrhiza glabra L. saponins modulate biophysical properties of bacterial model membranes and affect their interactions with tobramycin

Submitted to

Langmuir Journal

by

Adam Grzywaczyk [1], Monika Rojewska [1], Wojciech Smułek[1], Daniel A. McNaughton[2,3], Krystyna Prochaska [1], Philip A. Gale[2,3], Ewa Kaczorek [1]*

[1]Institute of Chemical Technology and Engineering, Faculty of Chemical Technology, Poznan University of Technology, ul. Berdychowo 4, 60-965 Poznan, Poland

[2]School of Mathematical and Physical Sciences, Faculty of Science, University of Technology Sydney, PO Box 123, Broadway, NSW, 2007, Australia

[3]School of Chemistry, The University of Sydney, NSW 2006, Australia.

Corresponding Author:

Adam Grzywaczyk Institute of Chemical Technology and Engineering, Faculty of Chemical Technology, Poznan University of Technology, ul. Berdychowo 4, 60-965 Poznan, Poland e-mail: adam.grzywaczyk@doctorate.put.poznan.pl; phone: +48 616653686

E-Supplementary data

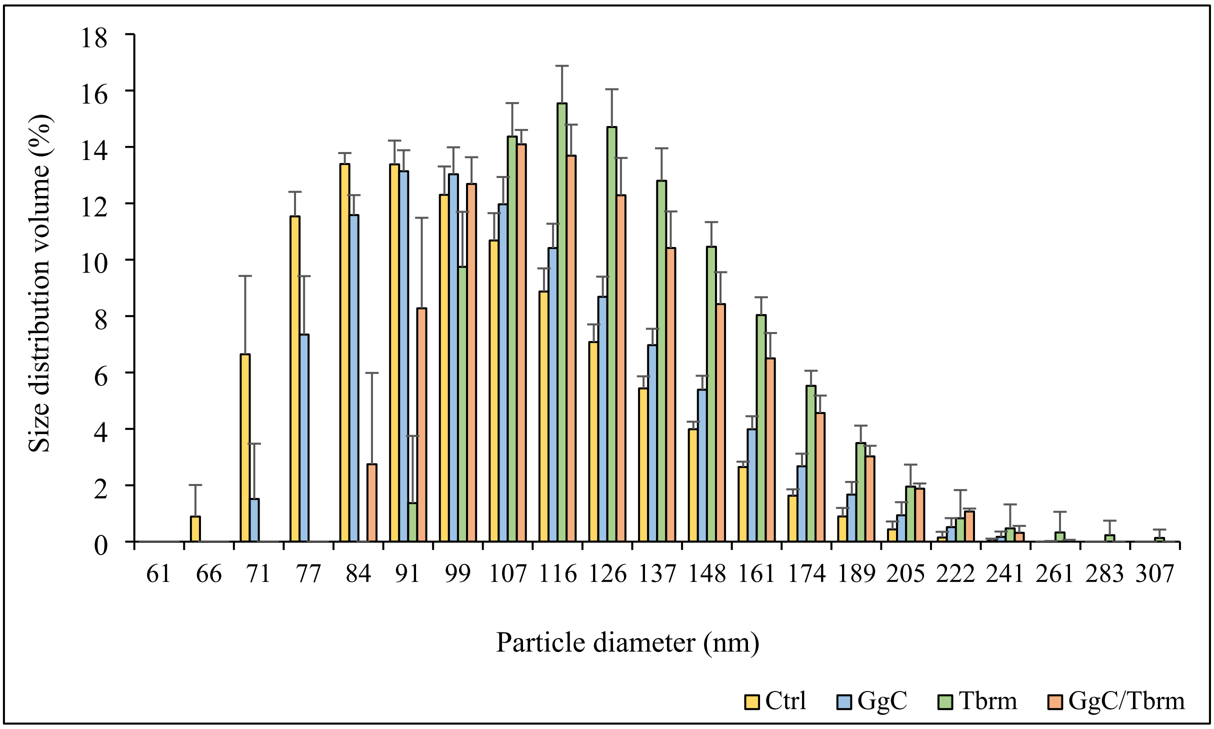

S1. Vesicle volume size distribution affected by *Glycyrrhiza glabra* L. root extract (GgC) and tobramycin. Ctrl - untreated sample, GgC - 5 mg/L treated, Tbrm - 10 mg/L Tobramycin, GgC/Tbrm 5 mg/L, GgC + 10 mg/L of Tobramycin effect

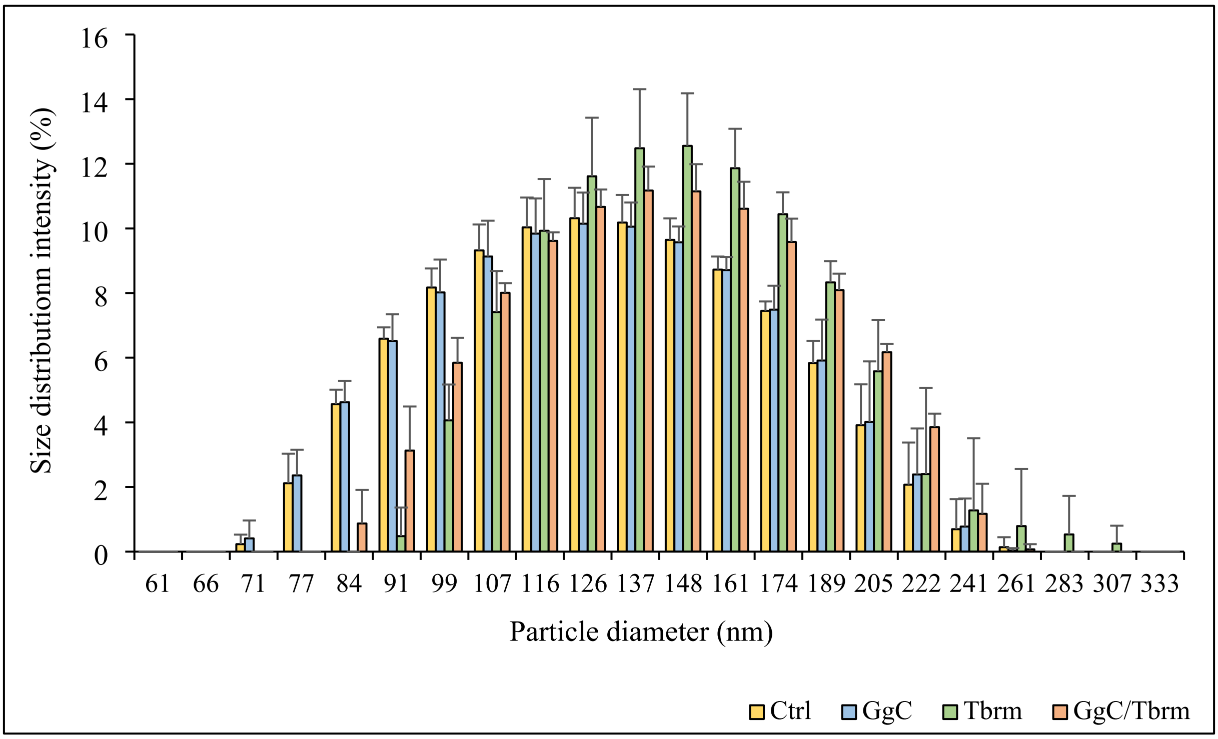

S2. Vesicle intensity size distribution affected by *Glycyrrhiza glabra* L. root extract (GgC) and tobramycin. Ctrl - untreated sample, GgC - 5 mg/L treated, Tbrm - 10 mg/L Tobramycin, GgC/Tbrm 5 mg/L, GgC + 10 mg/L of Tobramycin effect

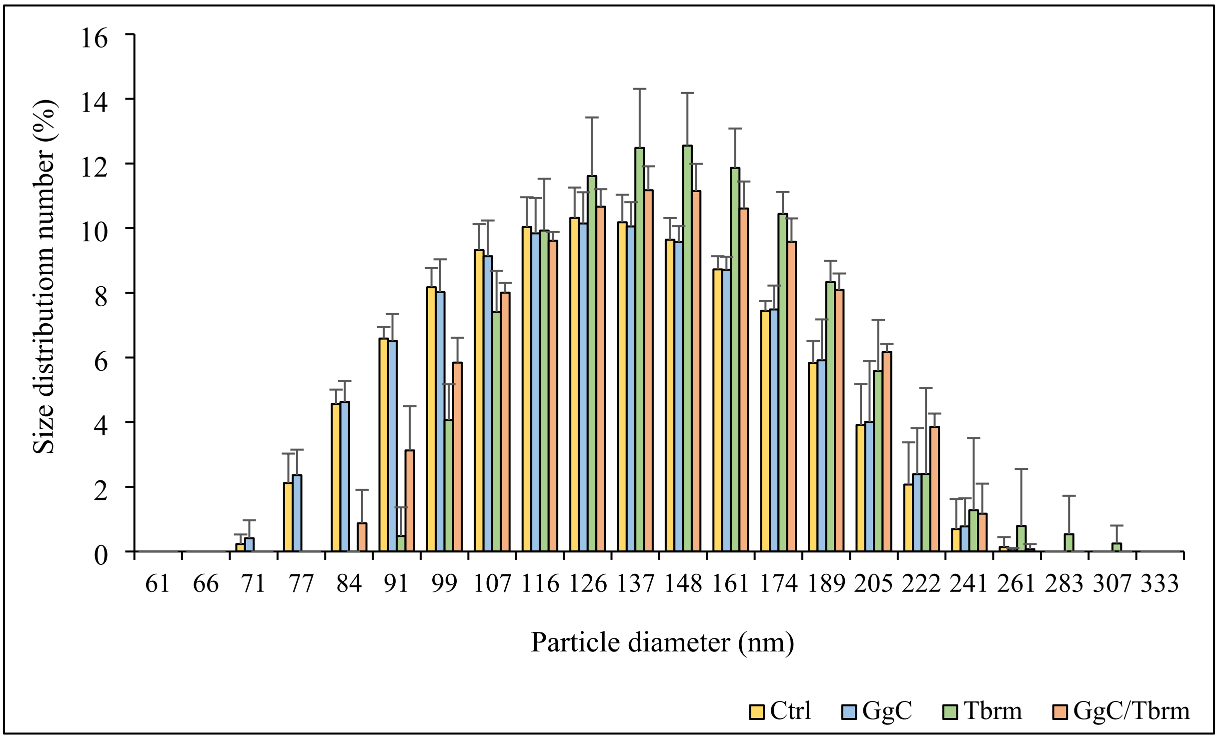

S3. Vesicle number size distribution affected by *Glycyrrhiza glabra* L. root extract (GgC) and tobramycin. Ctrl - untreated sample, GgC - 5 mg/L treated, Tbrm - 10 mg/L Tobramycin, GgC/Tbrm 5 mg/L, GgC + 10 mg/L of Tobramycin effect

Dynamic light scattering (DLS) analysis can represent particle size distribution according to three distinct weighting models: number, volume, and intensity. To facilitate clarity and comprehensive interpretation, all three distribution models are provided here, in the supplementary material. Each of these representations offers valuable insights, yet the selection of an appropriate model depends on the specific research questions or experimental requirements being addressed.

The volume-based distribution illustrates the size distribution based on the total volume occupied by particles. This distribution provides a balanced representation, moderately sensitive to both small and large particles, and thus often serves as a good general representation of sample heterogeneity. Intensity-based distribution, on the other hand, emphasises larger particles, as intensity scales strongly (proportionally to the sixth power) with particle size. Consequently, even a small number of large particles can dominate the intensity profile. This distribution is particularly useful for identifying aggregation or the presence of larger contaminants within the sample. The number-based distribution, reflects the actual numeric proportion of particles present, emphasising smaller particles, which are typically the most numerous in colloidal systems. However, this distribution can, on occasion, result in an underestimation of the significance of larger particles due to their low count.
